# Transarterial Chemoembolization (TACE) plus Sorafenib Versus TACE for Intermediate or Advanced Stage Hepatocellular Carcinoma: A Meta-Analysis

**DOI:** 10.1371/journal.pone.0100305

**Published:** 2014-06-19

**Authors:** Leida Zhang, Peng Hu, Xi Chen, Ping Bie

**Affiliations:** Department of Hepatobiliary Surgery, Southwest Hospital, Third Military Medical University, Chongqin, People’s Republic of China; Xiangya Hospital of Central South University, China

## Abstract

**Background:**

Sorafenib is used in patients with intermediate or advanced stage hepatocellular carcinoma (HCC) before or after of transarterial chemoembolization (TACE). However, the survival outcomes of TACE combined with sorafenib versus TACE alone remain controversial. Thus, we conducted a meta-analysis to evaluate the efficacy and safety of the combination therapy of TACE plus sorafenib in patients with intermediate or advanced stage of HCC.

**Methods:**

Pubmed and Embase databases were systematically reviewed for studies published up to November 2013, that compared TACE alone or in combination with sorafenib. Pooled hazard ratios (HRs) with 95% confidence intervals (95%CIs) were calculated for overall survival (OS), time to progression (TTP), objective response rate (ORR), and progression free survival (PFS) using random-effects or fixed-effects model, depending on the heterogeneity between the included studies.

**Results:**

Six studies published from 2011 to 2013, with a total of 1254 patients, were included in this meta-analysis. The pooled results showed that TACE combined with sorafenib significantly improved OS (HR = 0.65; 95% CI: 0.47–0.89, P = 0.007), TTP (HR = 0.68; 95% CI: 0.52–0.87, P = 0.003), ORR (HR = 1.06; 95% CI: 1.01–1.12, P = 0.021), but did not affect PFS (HR = 0.84; 95% CI: 0.62–1.14, P = 0.267). The incidence of grade III/IV adverse reaction was higher in the TACE plus sorafenib group than in the TACE group.

**Conclusions:**

The meta-analysis confirmed that the combination therapy of TACE plus sorafenib in patients with intermediate or advanced stage of HCC, can improve the OS, TTP, and ORR. This combination therapy was also associated with a significantly increased risk of adverse reactions.

## Introduction

Hepatocellular carcinoma (HCC) is the fifth most common cancer and the third leading cause of cancer death worldwide [1]. HCC results in over 650,000 deaths per year in the world, three-quarters of which occur in East Asian countries [2], [3]. Although surgical resection has been considered as definitive treatment for HCC, complete resection is not suitable for all patients because the disease is usually in its advanced stage when diagnosed [4]–[6].

Transarterial chemoembolization (TACE) is the standard therapy for HCC patients who are not suitable for surgical treatment [7]. TACE concentrates on chemotherapeutic agents at the tumor site while blocking the primary artery from feeding the tumor [8]. Thus, TACE is widely used to prolong the survival of patients with HCC. However, this procedure can stimulate local angiogenic factors that facilitate tumor regrowth and increase the possibility of metastasis [9].

Sorafenib, as a multikinase inhibitor that targets vascular endothelial growth factor (VEGFR), platelet-derived growth factor receptor (PDGFR), and Raf signaling, can block tumor growth and neoangiogenesis [10]. Sorafenib can target TACE-induced angiogenic factors and potentially enhance its efficacy [11]. Therefore, the addition of sorafenib to TACE in the treatment of HCC patients sounds reasonable.

In a randomized controlled clinical trial (RCT), the combination therapy of TACE plus sorafenib significantly improved the time to progression (TTP) [21]. However, the result was not observed in another RCT [20]. Therefore, we conducted a meta-analysis based on clinical trials to assess the efficacy and safety of TACE plus sorafenib in patients with intermediate or advanced stage of HCC.

## Materials and Methods

### Literature Search

A comprehensive search was performed to identify all published studies of TACE plus sorafenib in HCC patients. Pubmed and Embase database were searched before November 2013. Search terms were as follows: “sorafenib,” “nexavar,” “Thesaurus,” “carcinoma, hepatocellular,” “hepatocellular carcinoma,” “hepatomas,” “liver carcinoma,” “hepatocarcinoma,” “liver cell carcinoma,” “liver cancer,” “transarterial chemoembolization,” “TACE”. The reference lists of retrieved articles were also screened until no potential articles can be found.

### Review Strategy

Endnote bibliographic software was used to create an electronic library of citations identified in the literature search. Both Pubmed and Embase searches were performed using Endnote; duplicate records were deleted. Two independent investigators were (Leida Zhang and Peng Hu) trained to perform the abstract review and full text review thereafter. Disagreements between the two investigators were resolved by consensus and discussion. A standardized data extraction form was used for data extraction. The following data from the included studies were extracted: lead author; number of patients (TACE plus sorafenib/control); baseline patient characteristics; treatment; study region; primary endpoint; secondary endpoint; hazard ratio(HR) with 95% confidence interval(CI) for OS, TTP, and PFS; and number of adverse events in both TACE plus sorafenib group and TACE group.

### Study Inclusion and Exclusion Criteria

We included studies based on the following inclusion criteria: (1) HCC patients treated with TACE were assigned to sorafenib group or control group; (2) Data of efficacy and/or safety analyses were reported. Comments, editorials, systematic reviews or studies unrelated with our topics were excluded from final analysis. No publication language was limited.

### Quality Assessment

A modified Newcastle-Ottawa scale was used to assess the quality of nonrandomized studies included in this meta-analysis [12]. The scale consists of three items that describe patient selection, comparability of the TACE plus sorafenib and TACE placebo/alone groups, and outcome assessment. The quality scale ranged from 0 to 9 points. Articles with ≥6 points were considered as high quality.

### Statistical Analysis

We assessed the overall efficacy of TACE plus sorafenib in the treatment of HCC patients based on the data from the studies included. For the time-to-event variables [i.e., overall survival (OS), time to progression (TTP) and progression free survival (PFS)], HRs with 95%CI were directly extracted or calculated by a calculation sheet as previously described [13]. The incidences of treatment-related adverse events were treated as dichotomous variables, and the number of adverse events and total number of patients were extracted from the included studies. Afterward, the risk ratio (RR) with 95% CI was calculated. Pooled estimates of HR or RR were calculated using the fixed-effects model (Mantel-Haenszel method) [14]. When substantial heterogeneity existed, the random-effects model (DerSimonian-Laird method) [15] was used to summarize the pooled data. A test for heterogeneity, defined as variation between individual trials for a given treatment rather than that expected from chance, was used to assess whether or not the magnitude of a given treatment effect varies between the trials. *I^2^* statistic describes the percentage of total variation across studies that is due to heterogeneity rather than chance. Studies with an *I* ^2^ value of <25%, ∼50%, ∼75%, and ∼100% were considered to have no, low, moderate, and high heterogeneity, respectively [16].

The presence of publication bias was evaluated using the Begg’s and Egger’s tests [17], [18]. A *P* value less than 0.05 was judged as statistically significant. All statistical analyses were performed using STATA version 12.0 (Stata Corporation, College Station, TX, USA).

## Results

### Identification of Eligible Studies

**Figure 1:**
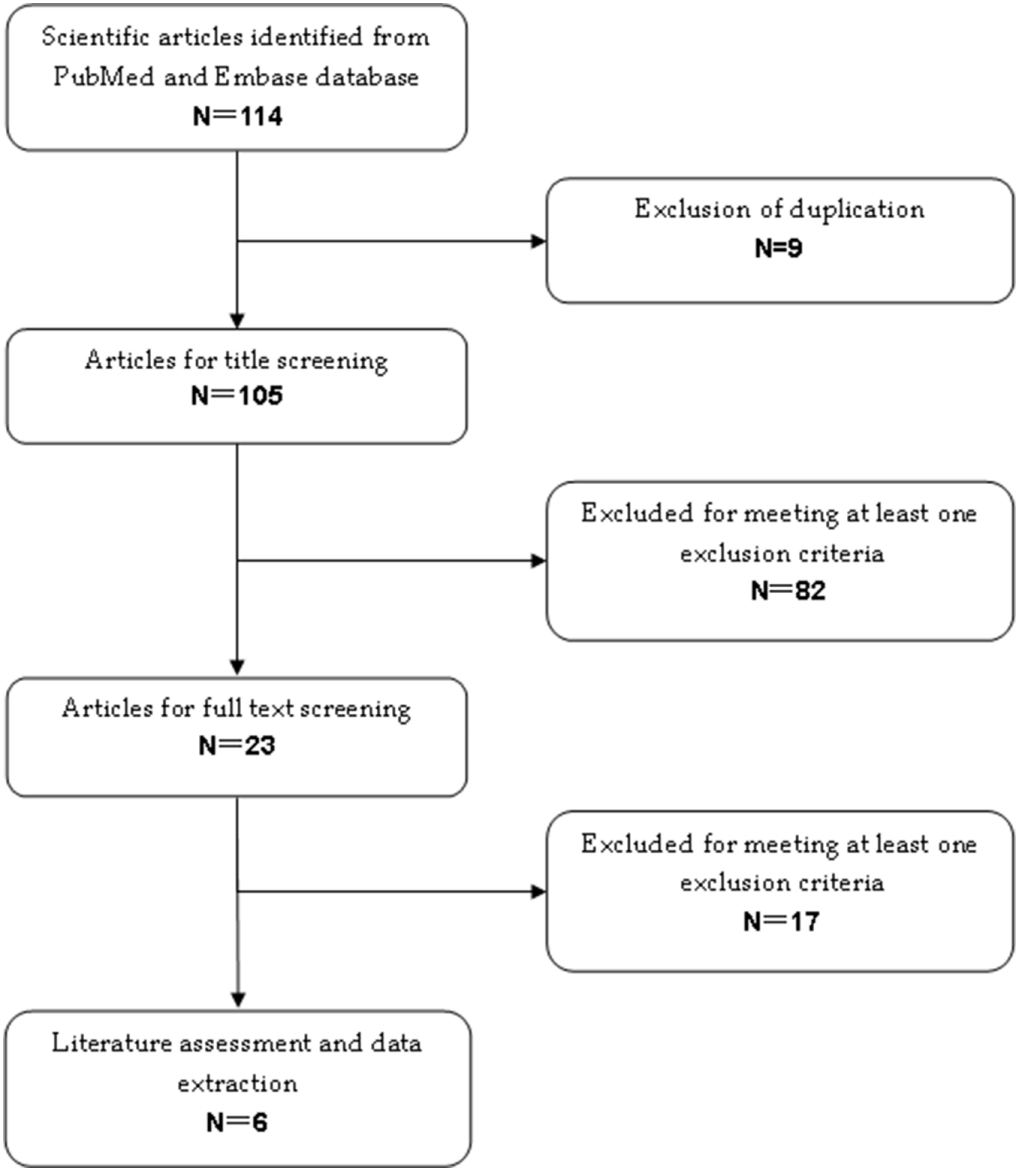
Eligibility of studies for inclusion in the meta-analysis.

The initial literature search identified 114 HCC-related citations from Pubmed and Embase databases. After excluding duplicate records, 82 and 17 studies were excluded after screening the abstract and the full text, respectively (Figure 1). Finally, six studies (n = 1254 patients) that met the inclusion criteria were included in the meta-analysis [19]–[24]. Among the six included studies, two were randomized controlled trials, two were propensity score-matched cohort studies, and two were retrospective cohort studies.

### Characteristics of Eligible Studies

The baseline characteristics of the six trials included in this meta-analysis are presented in Table 1. The six studies were published between 2011 and 2013. Among the studies included, two were conducted in China [19], [24], two in Korea [20], [23], one in Japan [20], one in Germany [21] and one in USA [22]. All studies included both men and women. All HCC patients enrolled in these trials met the following criteria: Child-Pugh class A cirrhosis, Eastern Cooperative Oncology Group (ECOG) performance status (PS) 0 or 1, adequate liver function and renal function. The judgment of treatment-related adverse events was assessed by the National Cancer Institute Common Terminology Criteria for Adverse Events (CTCAE) version 3.0. According to the eligibility criteria of the included studies, all the patients were definitely diagnosed to have intermediate or advanced HCC. The detailed eligibility criteria of these included studies are described in Table 1.

**Table 1:** Baseline Characteristics of the included studies.

Total (1254)	Median age(range)	Male/Female	Treatment	Region	Child-PughClass	ECOG PS	Virology	Primaryendpoint	Secondaryendpoints
Wei BAI,et al. [19](246)	54±13	73/9	TACE +SOR400 bid	China	Class A/B:63/19	PS 0/1/2/3/4:30/38/12/1/1	HBV/HCV/no infection:72/4/6	OS	TTP,DCR
	52±12	146/18	TACE		Class A/B:115/49	PS 0/1/2/3/4:48/101/15/0/0	HBV/HCV/No infection:147/7/10		
Masatoshi Kudo, et al. [20](458)	69	174/55	TACE +SOR400 bid	Japan +Korea	Class A 229	PS 0/1∶201/28	Alcohol/HBV/HCV/Other/LC:19/47/139/16/159	TTP	OS
	70	168/61	TACE +placebo		Class A 229	PS 0/1∶202/27	Alcohol/HBV/HCV/Other/LC:12/52/148/11/154		
Domenico Sansonno,et al. [21](62)	73±4	18/13	TACE +SOR400 bid	Germany	Class A 31	PS 0/1∶26/5	HCV 31	TTP	
	72.8±6.4	19/12	TACE + placebo		Class A 31	PS 0/1∶24/7	HCV 31	OS, PFS	
Adnan Muhammad, et al. [22](43)	61.4±7.5	13/0	TACE + SOR 200bid, thenincreasedto 400 bid		Class A/B:11/2	NR	Alcohol/HCV/Alcohol+ HCV/other:2/6/3/2		
	59.2±7.4	30/30	TACE	USA	Class A/B:23/7		Alcohol/HCV/other/Alcohol+ HCV/:1/17/1/11		
Gwang Hyeon Choi,et al. [23](355)	52 (26–75)	139/25	TACE +SOR400 bid	Korea	Class A/B:119/45	NR	HBV/HCV/HBV+HCV/Other:139/9/1/15	TTP,OS	
	54 (22–84)	166/25	SOR		Class A/B:136/55		HBV/HCV/HBV+HCV/Other:166/6/1/18		
Xu-Dong Qu,et al. [24](90)	51±11.7	41/4	TACE +SOR400 bid	China	Class A/B:33/12	PS 0/1∶43/2	NR	OS	
	49±11.0	41/4	TACE		Class A/B: 35/10	PS 0/1∶41/4	NR		

TACE, transartialchemoembolization; LC, liver cirrhosis; OS, overall survival; TTP, time to progression; DCR, disease control rate; NR, not report; HBV, hepatitis B virus; HCV, hepatitis C virus; SOR, sorafenib.

### Quality of the Included Studies

The quality of the nonrandomized studies was assessed by the Newcastle-Ottawa Scale (NOS), and the scores ranged from 8–9, indicating that these studies have high quality (Table 2).

**Table 2:** Newcastle-Ottawa Scale (NOS) for assessing the quality of nonrandomized trials.

Non RCTstudies	Selection			Comparability			Assessment ofoutcome			Total qualityscore
Author	Representativeness oftreated arm	Selection of comparativetreatment arm	Ascertainment oftreatment regimen	Demonstration that theoutcome of interest wasnot present atstart of study	Comparability betweenpatients in differenttreatment arms-main factor:Child-Pugh Class	Comparability betweenpatients in differenttreatment arms-secondaryfactor: Aetiology	Assessment of outcomewith independency	Adequacy of followup length	Lost to follow upacceptable (less than10% and reported)	
Wei Bai	*	*	*	*	*		*	*	*	8
AdnanMuhammad	*	*	*	*	*	*	*	*	*	9
GwangHyeon Choi	*	*	*	*	*	*	*	*		8
Xu-DongQu	*	*	*	*	*	*	*	*		8

### Overall Survival

**Figure 2:**
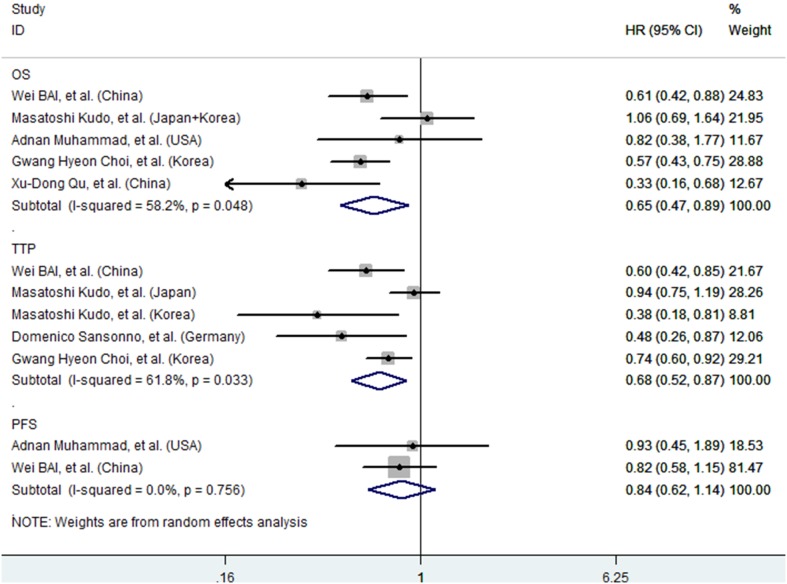
Overall survival (OS), time to progression (TTP), and progression.

Among the six studies included in the meta-analysis, five reported the results of OS rate [19], [20], [22]–[24]; wherein, two studies [20], [22] showed that the OS rate was similar between the two groups, whereas the remaining three studies [19], [23], [24] demonstrated that the OS was significantly better in the TACE combined with sorafenib than in the TACE group. The pooled HR for the OS in the included studies performed using the random- effects was 0.65 (95% CI: 0.47–0.89; P = 0.007; *I* ^2^ = 58.2%, P = 0.048). This value represents a 35% reduction in the risk of death in patients treated with TACE combined with sorafenib (Figure 2). We performed a sensitivity analysis to examine the potential source of heterogeneity. Similar result was obtained (HR = 0.66; 95% CI: 0.54–0.81; P = 0.0054) when three trials with a modest size (N≤100) were excluded. The overall estimate did not substantially change (HR = 0.70; 95% CI: 0.49–0.99; P = 0.0043) when the two retrospective cohort studies were excluded. The Egger’s test (P = 0.971) and Begg’s test (P = 0.806) revealed no publication bias.

### Time to Progression

Four of the six studies included in the meta-analysis presented data on TTP [19]–[21], [23], among which, two [20], [21] were randomized controlled studies and two [19], [23] were propensity score-matched cohort studies. Among these four trials, one showed that the HCC patients treated with TACE had similar benefit in TTP compared those treated with TACE combined with sorafenib, whereas the remaining three trials indicated that TTP was significantly better in patients treated with TACE plus sorafenib group than in those treated with TACE alone. The pooled HR for the TTP in the four trials was 0.68 (95% CI: 0.52–0.88; P = 0.003; *I* ^2^ = 61.8%, P = 0.0465), indicating a 32% reduction in the risk of TTP in the HCC patients treated with TACE combined with sorafenib (Figure 2). After exclusion of the non-randomized controlled trials, the pooled HR for TTP was 0.71 (95% CI: 0.59–0.85; P = 0.001). The Egger’s test (P = 0.078) and Begg’s test (P = 0.086) showed no publication bias.

### Progression Free Survival

PFS data were provided in two trials [19], [22]. Both trials showed that the treatment of TACE combined with sorafenib did not improve the PFS in patients with intermediate or advanced stage of HCC compared with those treated with TACE alone. The pooled HR for PFS in the two non-randomized controlled trials was 0.84 (95% CI: 0.62–1.14; P = 0.267; *I* ^2^ = 0.0%, P = 0.756) (Figure 2). Publication bias analysis was not performed, because the number of included studies was less than 5.

### Objective Response Rate

**Figure 3:**
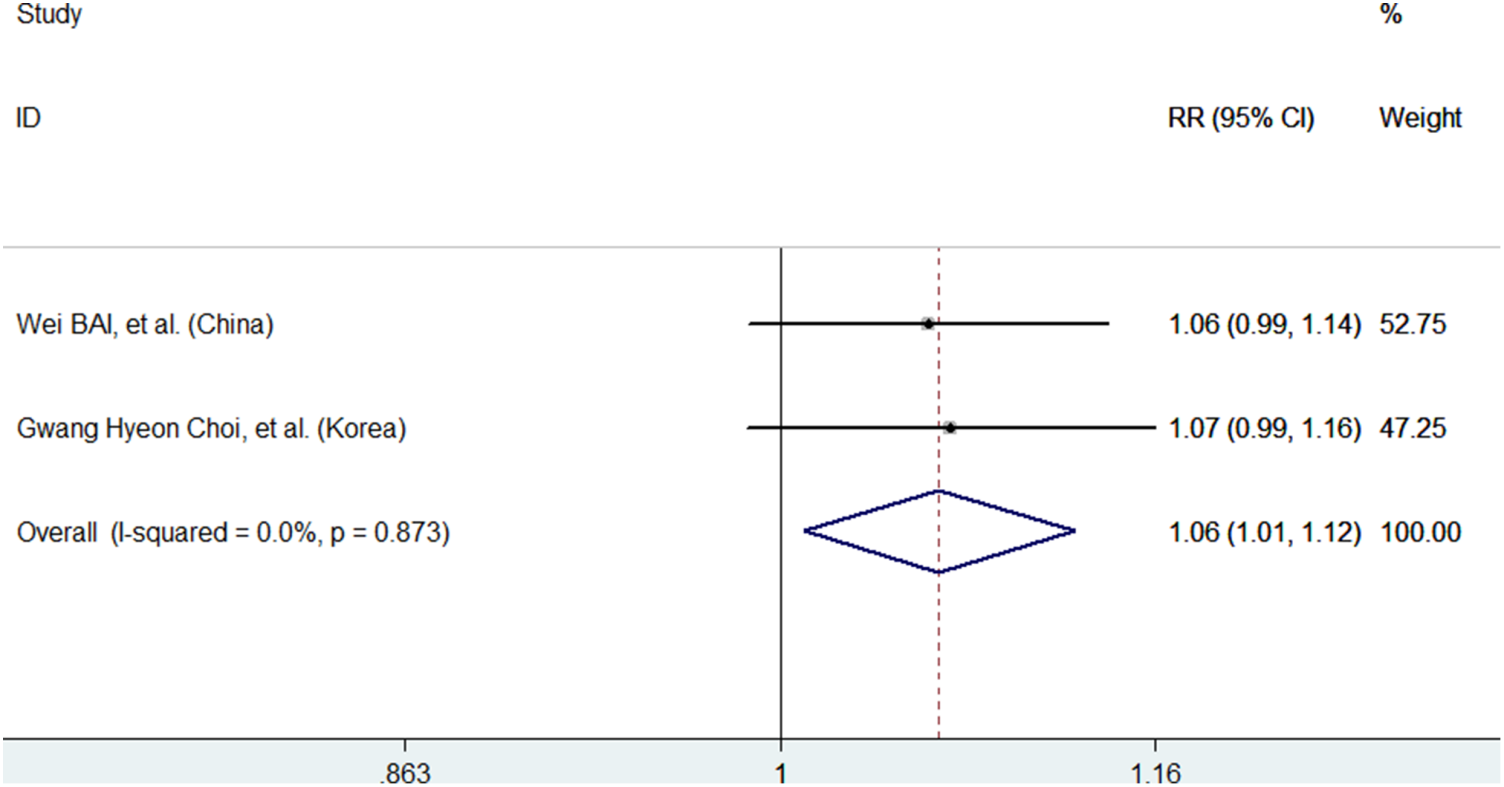
Objective response rate (ORR) for the combination of TACE plus sorafenib with TACE.

Two trials [19], [23] presented data on ORR. The two propensity score-matched cohort trials, showed that the pooled RR for ORR was 1.06 (95% CI: 1.01–1.12; P = 0.021; *I* ^2^ = 0.0%, P = 0.873), indicating that the HCC patients treated with TACE plus sorafenib had a better response than those treated with TACE alone (Figure 3). Publication bias analysis was not performed because only two studies were included.

### Adverse Reactions

All six included studies reported the occurrence of adverse reactions, including hand-foot skin reactions, diarrhea, fatigue, gastrointesestinal, hypertension, rash or desquamation, abdominal pain, nausea, vomiting, hepatic encephalopathy, and elevated lipase/AST/ALT.

However, only three studies provided available data for analysis. The pooled estimate calculated based on the fixed-effects model showed that, the incidence rates of grade-III/IV hand-foot skin.

**Figure 4:**
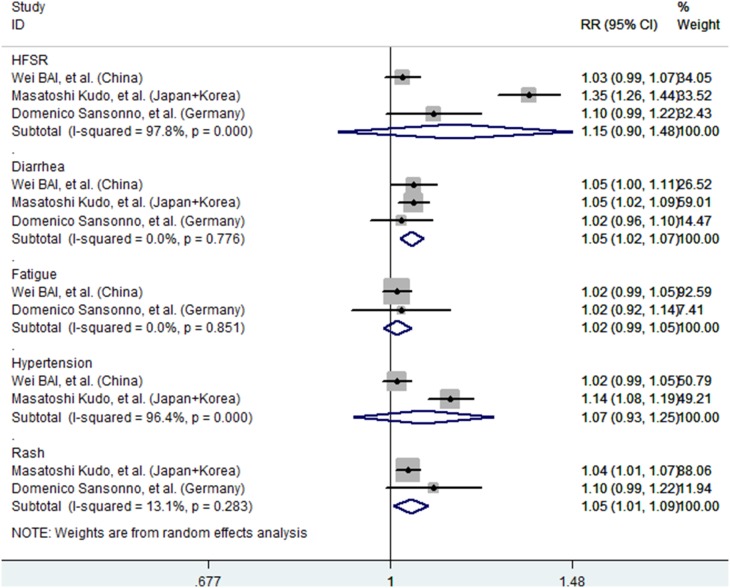
Relative risk of Grade III/IV adverse events in patients treates with TACE plus sorafenib versus TACE.

Reactions (RR = 1.22, 95%CI: 1.17–1.27; P = 0.00; *I* ^2^ = 97.8%, P = 0.00), diarrhea (RR = 1.05, 95%CI: 1.02–1.08; P = 0.00; *I* ^2^ = 0.0%, P = 0.776), hypertension (RR = 1.10, 95%CI: 1.06–1.13; P = 0.00; *I* ^2^ = 96.4%, P = 0.00), rash or desquamation (RR = 1.05, 95%CI: 1.02–1.08; P = 0.00; *I* ^2^ = 13.1%, P = 0.28) were higher in TACE plus sorafenib group than that in TACE group (Figure 4).

## Discussion

To the best of our knowledge, this meta-analysis is the first to assess the efficacy and safety of the combination treatment of TACE plus sorafenib in patients with intermediate or advanced stage of HCC. The present meta-analysis from two randomized controlled trials and four cohort studies, provided relatively high level of evidence, showing that HCC patients treated with TACE combined with sorafenib had significantly higher OS (HR = 0.64; 95%CI: 0.47–0.89), TTP (HR = 0.68; 95%CI: 0.52–0.88) and ORR (HR = 1.06; 95%CI: 1.01–1.12) than those treated with TACE alone, but with a well tolerated grade-III/IV adverse reactions. Our findings demonstrated the significant benefits of TACE plus sorafenib in terms of survival and complications.

Sorafenib, as a multikinase inhibitor, delays the tumor progression in HCC patients by inhibiting tumor cell proliferation and neoangiogenesis [25], [26]. Local treatments including TACE, surgery, or radiofrequency ablation, can induce the overproduction of vascular endothelial growth factor (VEGF), which may promote disease progression or metastasis [9], [27]. Therefore, sorafenib, as complementary treatment acting on VEGF, may enhance the treatment outcomes by reducing VEGF overexpression, when administrated before or sequential use of TACE.

The clinical benefits of the abovementioned concept have been demonstrated in five of the studies included in this meta-analysis [19], [21]–[24]. However, in the phase III randomized, controlled trial [20] conducted by Masatoshi Kudo, et al., sorafenib did not significantly improve TTP or OS by central review in Japan and Korea patients who responded to TACE. Their results showed that the median TTP values in the sorafenib and placebo group were 5.4 and 3.7 months, respectively (HR = 0.87; 95%CI: 0.70–1.09) [20]. The subgroup analyses suggested that several factors may have potential effects on TTP, including age, treatment lag, treatment duration, number of prior TACE course, administration dose, and nationality. Patients from Korea had significantly higher TTP (HR = 0.38; 95%CI: 0.18–0.81) than those from Japan (HR = 0.94; 95%CI: 0.75–1.19). Interestingly, Korean patients featured younger age, higher rate of HBV infection, higher response rate to TACE, less tumor burden, and short treatment lag than the Japanese patients [20]. Moreover, the median duration of sorafenib treatment was noticeably longer (30.9 weeks vs 16.1 weeks) in Korean patients than in Japanese patients [20]. The prolonged time between TACE and sorafenib treatment, as well as the duration of sorafenib administration may have contributed to the suboptimal outcome. Thus, further investigation is urgently needed to address this issue.

In this meta-analysis, we found that the treatment-associated adverse events were mostly mild to moderate. Common adverse events, including hand-foot syndrome, diarrhea, hypertension, and rash or desquamation, were encountered in the combination treatment group, with RR values of 1.22, 1.05, 1.10, and 1.05, respectively. Our results were similar to those of previous meta-analysis studies [28], [29]. Despite being recognized as common side effects, these events significantly affect the therapeutic compliance of the patients. Dose reduction or pauses in sorafenib treatment may hamper the attainment of therapeutic benefits in the combined group. Thus, different treatment strategies should be recommended in terms of decreased dose and treatment schedules for such patients.

Randomized and nonrandomized studies have different results, and differences between these studies exist in many directions [30]. In our meta-analysis, the pooled estimates in both meta-analysis of RCTs and non-RCTs were similar, although these studies may have potential biases in varying study designs. This fact adds robustness to the validity of our results. At the time of our writing, several phase II/III trials aiming to assess the efficacy of TACE combination with sorafenib in patients with intermediated or advanced HCC are in progress. The preliminary report from published abstracts showed that the outcome of the combination therapy was promising [31]–[34].

Several potential limitations in this meta-analysis should be considered when interpreting our results. First, among the six studies included, only two were RCTs and the remaining four were cohort studies. Although the cohort studies can reflect the “real-world” and further support the conclusion, cohort data are of course inclined to bias because of the patient selection. Thus, physicians should carefully interpret our results when applying them in clinical practice. Second, the characteristics of population (age, cause of liver disease, vascular invasion, and previous therapy), the sorafenib regimen (dosage, treatment lag, and treatment duration), and study designs vary considerably between the included trials. These factors may increase the heterogeneity and affect the results. Third, half of the included studies have small sample size that may lead to an overestimation of the treatment effects. Furthermore, because of the limited number of studies regarding the interest outcomes, caution should be taken when interpreting the results. Finally, performing a more detailed subgroup analysis based on the disease status of patients is difficult because of the presence of heterogeneity among the studies enrolled in each study.

In summary, the current meta-analysis suggests that the combination therapy of sorafenib plus TACE can significantly improve OS, TTP, and ORR for patients with intermediate or advanced HCC, with tolerable toxicity. The use of TACE plus sorafenib for HCC treatment of HCC is promising. However, considering the heterogeneity among study designs and small-scale RCTs, further multi-centre, well-designed RCTs are needed to verify these findings and investigate the factors affecting the survival outcomes.

## Supporting Information

Checklist S1 PRISMA Checklist.
(DOC)
